# Multiple Quantum Coherences Hyperpolarized at Ultra‐Low Fields

**DOI:** 10.1002/cphc.201900757

**Published:** 2019-10-17

**Authors:** Kai Buckenmaier, Klaus Scheffler, Markus Plaumann, Paul Fehling, Johannes Bernarding, Matthias Rudolph, Christoph Back, Dieter Koelle, Reinhold Kleiner, Jan‐Bernd Hövener, Andrey N. Pravdivtsev

**Affiliations:** ^1^ High-Field Magnetic Resonance Center Max Planck Institute for Biological Cybernetics Max-Planck-Ring 11 72076 Tübingen Germany; ^2^ Department for Biomedical Magnetic Resonance University of Tübingen Hoppe-Seyler-Str. 3 72076 Tübingen Germany; ^3^ Institute for Biometrics and Medical Informatics Otto-von-Guericke University Building 02 Leipziger Str. 44 39120 Magdeburg Germany; ^4^ Physikalisches Institut and Center for Quantum Science (CQ) in LISA^+^ University of Tübingen Auf der Morgenstelle 14 72076 Tübingen Germany; ^5^ Section Biomedical Imaging Molecular Imaging North Competence Center (MOIN CC) Department of Radiology and Neuroradiology University Medical Center Kiel Kiel University Am Botanischen Garten 14 24114 Kiel Germany

**Keywords:** hyperpolarization, multiple quantum coherence, parahydrogen, SABRE, SQUID

## Abstract

The development of hyperpolarization technologies enabled several yet exotic NMR applications at low and ultra‐low fields (ULF), where without hyperpolarization even the detection of a signal from analytes is a challenge. Herein, we present a method for the simultaneous excitation and observation of homo‐ and heteronuclear multiple quantum coherences (from zero up to the third‐order), which give an additional degree of freedom for ULF NMR experiments, where the chemical shift variation is negligible. The approach is based on heteronuclear correlated spectroscopy (COSY); its combination with a phase‐cycling scheme allows the selective observation of multiple quantum coherences of different orders. The nonequilibrium spin state and multiple spin orders are generated by signal amplification by reversible exchange (SABRE) and detected at ULF with a superconducting quantum interference device (SQUID)‐based NMR system.

## Graphical abstract

**Ultra‐low field COrrelation SpectroscopY (ULF COSY)**: Hyperpolarized homo‐ and heteronuclear (^1^H‐^19^F) multiple quantum coherences (MQCs) from zero to the third order were observed at 91 μT by SQUID based NMR. MQCs were assigned by using density matrix simulations and measured here by a phase cycling modified ULF COSY sequence. The method allowed us to look deeper inside hyperpolarization processes at low magnetic fields.

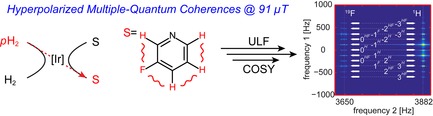

## 1 Introduction

The hyperpolarization of nuclear spins and the associated breakthroughs in physics, chemistry, biology and medicine continue to inspire the work of many scientists around the world. During the past decades, various hyperpolarization techniques have been developed^1^, ^2^, ^3^, ^4^, ^5^, ^6^, ^7^, ^8^, ^9^, ^10^, ^11^, ^12^, ^13^, ^14^ to boost the magnetic resonance (MR) signal in order to bring new applications to industry^15^, ^16^, ^17^ and life‐sciences.^18^, ^19^, ^20^, ^21^, ^22^, ^23^, ^24^, ^25^ One of these methods is signal amplification by reversible exchange (SABRE),^26^, ^27^, ^28^, ^29^, ^30^, ^31^, ^32^, ^33^, ^34^, ^35^, ^36^ where parahydrogen (*p*H_2_) is used to polarize dissolved molecules by their mutual exchange with a transient complex. SABRE is unique in providing continuous hyperpolarization in the liquid state with high‐throughput^26^, ^37^ and is relatively cost‐efficient.

SABRE at high magnetic field for high‐resolution NMR encounters difficulties due to magnetic field inhomogeneity caused by *p*H_2_ bubbling;^38^ this is not a problem for ultra‐low field (ULF) NMR and even allows continuous SABRE,^39^ radio‐wave amplification by stimulated emission of radiation (RASER)^13^ and QUASi‐Resonance SABRE (QUASR).^40^

Much effort is being undertaken to bring SABRE to “life sciences”, but despite considerable efforts, a clean, highly polarized, highly concentrated biologically relevant contrast agent was not produced yet.^41^, ^42^, ^43^, ^44^, ^45^, ^46^

When it comes to biomedical applications, usually, it is the goal to populate one dedicated spin state and, as a result, boost the MRI signal of the targeted nuclei. Here, we report the opposite effect: we discovered that in SABRE experiments at low magnetic fields various multiple spin orders are hyperpolarized. It was revealed by simultaneous observation of hyperpolarized homo‐ (^1^H) and heteronuclear (^1^H‐^19^F) zero‐order and multiple quantum coherences (QCs), up to the third order. This confirms that redistribution of *p*H_2_ spin alignment in SABRE results not only in the substrate's magnetization but also in the population of multiple spin orders, including homo‐ and heteronuclear zz‐orders and singlet spin states.^27^, ^35^, ^36^, ^47^, ^48^, ^49^ This observation illustrates that the transfer of *p*H_2_ spin order to a substrate at the low magnetic field can be greatly improved by using more targeted polarization transfer techniques discussed elsewhere.^40^, ^50^

The redistribution of *p*H_2_ spin alignment and hyperpolarized multiple quantum coherences were revealed using a superconducting quantum interference device (SQUID)‐based ULF MR spectrometer, designed as a field‐cycling system, which operates in the magnetic field range of 10 μT to 20 mT. In this range of fields SQUID‐based magnetic field sensors^51^ and optical magnetometers^52^ become superior to conventional Faraday coils.^53^

**Figure 1:**
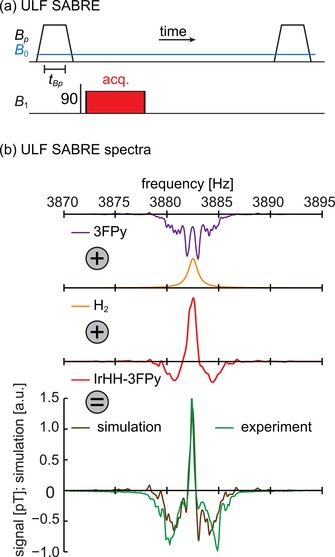
ULF SABRE scheme (a) and corresponding simulated and experimental spectra (b). After hyperpolarization at *B_p_*=5.2 mT and a 90° excitation pulse at *B*_0_=91 μT, the SABRE signal is acquired. Simulated ^1^H‐spectra of hyperpolarized 3FPy substrate (purple), H_2_ (orange) and IrHH‐3FPy complex (red). The weighted sum of these spectra (brown) was fitted to the experimental data (green). This data indicates that all three constituents of SABRE (H_2_, Ir‐complex and substrate) were hyperpolarized and observed.

The combination of this fast field‐cycling SQUID‐based MR system and the continuous generation of SABRE allows to observe hyperpolarized substrate, H_2_ and SABRE complexes (IrHH‐substrate) at ULF (Figure 1). Before, similar measurements were achieved only at much higher magnetic fields.^54^, ^55^ The superposition of several hyperpolarized species or states in the ^1^H ULF SABRE spectrum is evident (Figure 1b) and in the absence of chemical shift resolution the sign of polarization can serve as a contrast; at 91 μT the chemical shift difference of 1 ppm corresponds to only 4 mHz frequency variation.

In the main text, the findings and their evaluation will be demonstrated on 3‐fluoropyridine (3FPy), used as a substrate with the ubiquitous SABRE catalyst [IrIMesCODCl].^56^ Fluorinated variants of common SABRE substrates (3FPy and ethyl 5‐fluoronicotinate – see the Supporting Information) were used here to demonstrate the presence of homo‐ and heteronuclear quantum coherences.

## 2 Results and Discussion

### 2.1 ULF COSY Scheme

**Scheme 1:**
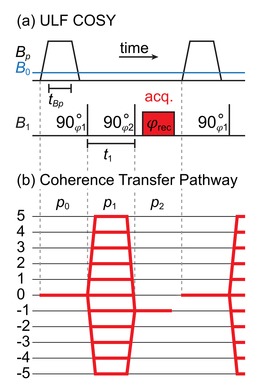
ULF COSY experiment (a) and the corresponding evolution of the quantum coherences, $p_{n}$ (b). The coherence selection pathway starts from different zero quantum coherences and multiplet spin orders with $p_{0}$ =0, a first $90_{\;}^{0}$ pulse converts these spin order into quantum coherences $p_{1}$ for a period of time, *t*_1_, (→*frequency* 1 domain) and after the second $90_{\;}^{0}$ pulse NMR signal observation starts (→*frequency* 2 domain), therefore only $p_{2}$ =−1 is retained (acq.‐block stands for signal acquisition).

To elucidate the hyperpolarized spin states, we modified a conventional correlation spectroscopy (COSY) pulse sequence (Scheme 1a) for ULF NMR^57^, ^58^ – ULF COSY, by adding a hyperpolarization phase for each *t*_1_ variation step and simultaneous excitation of ^1^H and ^19^F spins. Note that we also tried to implement the method with a variable excitation flip angle,^59^, ^60^, ^61^ however, in the given conditions it was shown to be unpractical (cf. Supporting Information, Section 4).

**Figure 2:**
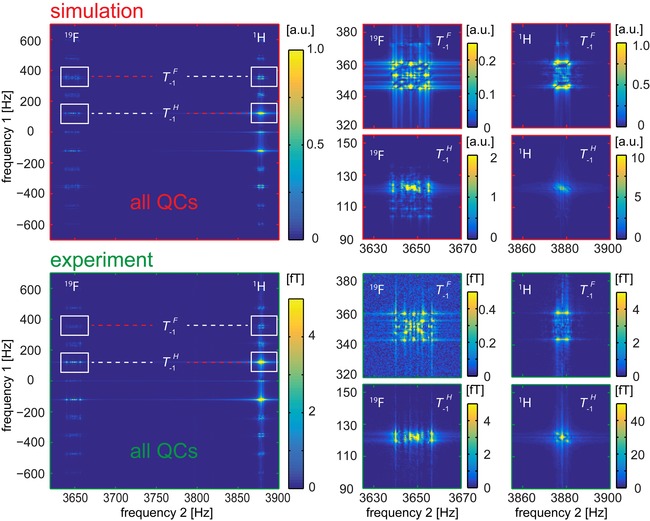
Hyperpolarized quantum coherences measured with the ULF COSY experiment (*B_p_*=5.2 mT and *B*_0_=91 μT): simulated (top) and experimental (bottom) amplitude spectra of 3FPy. $T_{y}^{x}$ is a symbol for QCs, where *y* is the order of the QC and *x* indicates the types of nuclei involved (Table S5). On the right, the zoomed out $T_{-1}^{H}$ and $T_{-1}^{F}$ QCs measured at ^1^H and ^19^F resonances are shown (indicated by the white rectangles). Red and white dashed lines mark the diagonal and off‐diagonal peaks, respectively. Up to third‐order coherences were observed. Simulations reproduced experimental details.

The ULF COSY sequence starts with a prepolarization phase at the magnetic field strength *B_p_* for the period of *t_Bp_* by means of SABRE. When the magnetic field was reduced to the observation field, *B*_0_, two $90_{\;}^{0}$ ^1^H‐^19^F excitation pulses with the phases $\varphi_{1}$ and $\varphi_{2}$ and a variable interpulse interval, *t_1_*, were applied. After the second 90° pulse, the signal was acquired with a receiver phase $\varphi_{rec}$ . A 2D Fourier transformation along the direct readout (→*frequency* 2) and the indirect “*t*_1_ direction” (→*frequency* 1) result in a 2D spectrum (Figure 2).

### 2.2 ULF COSY Spectrum

A relatively narrow spectral width (SW) of 4 kHz instead of the required 40 kHz in the indirect dimension was chosen to obtain a highly resolved 2D ULF COSY spectrum at a reasonable time (<8 h), however, as a result the signals of the quantum coherences folded into the bandwidth between −2 kHz and +2 kHz (Figure 2 and Table S5 in the Supporting Information). Such frequencies can be easily obtained using the product operator formalism (SI, Section 7).^62^

Four uncoupled undistinguishable ^1^H spins and one ^19^F spin in maximum have 26 different sorts of quantum coherences with different frequencies due to the Zeeman interaction (Table S5); a total number of coherences of 5 spin‐1/2 system is 2^4^(2^5^–1)=496. Although J‐coupling constants make the system more complex, at ULFs of 91 μT, the Zeeman interaction is still the leading term which defines the frequencies of quantum coherences and J‐coupling only adds multiplicity to the peaks (Figure 2).

These coherences can be encoded by ULF COSY during the time period *t*_1_ and observed as separate peaks in a 2D spectrum. By chance, at the given magnetic field, $B_{0}$ ≈91 μT, and SW=4 kHz, some QCs have the same (aliased) frequencies and only 15 peaks were clearly separable in the simulations. Experimentally only 11 peaks were obtained (Figure 2a and Figure S5 in the Supporting Information). These peaks belong to QCs from zero up to the third order.

This demonstrates that not only one and two‐spin orders like, ${\hat{I}}_{kZ}$ , and ${\hat{I}}_{kZ}{\hat{I}}_{mZ}$ are polarized by means of SABRE but also 3‐spin orders like ${\hat{I}}_{kZ}{\hat{I}}_{mZ}{\hat{I}}_{nZ}$ are populated [see SI, Eq. (S3)]. The signal intensity of predicted higher‐order QCs (fourth and fifth) was below the noise level of the setup.

**Figure 3:**
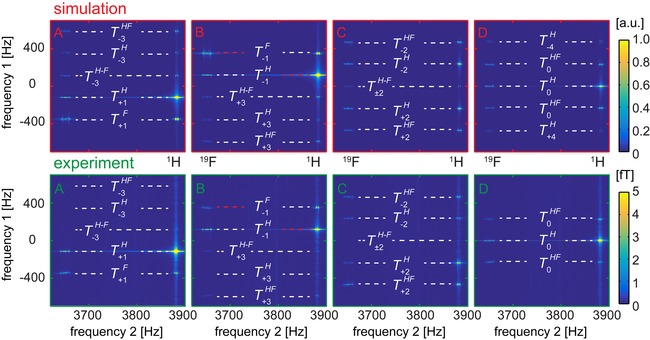
Separation of QCs in the coherence selective ULF COSY experiment (*B_p_*=5.2 mT and *B*_0_=91 μT): simulated (top) and experimental (bottom) amplitude spectra of 3FPy obtained with four different phase alternating methods (here A−D corresponds to phase cycling schemes mentioned in the text and given in the SI, Table S4). $T_{y}^{x}$ is a symbol of QCs, where *y* is the order of the QC and *x* indicates the types of nuclei involved (SI, Table S5). The red and white dashed lines mark the diagonal and off‐diagonal peaks, respectively. ULF COSY spectra without phase cycling are shown in Figure 2.

**Figure 4:**
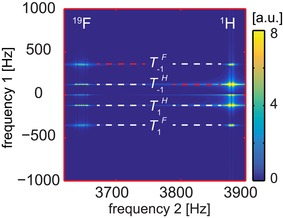
Simulated ULF COSY amplitude spectrum of 3FPy after polarization of longitudinal states alone (thermal Zeeman polarization of order *p*=0 and rank *l*=1). Note that in the case of SABRE higher‐order quantum coherences appear (Figures 2 and 3). The COSY spectrum with an initial longitudinal magnetization shows only first order (and zero H−H orders) quantum coherences. Also, note that the shapes of the peaks are different.

Note, that only two peaks are “diagonal” COSY peaks, which are the result of the “‐1” quantum coherence evolution during the *t*_1_ time interval and acquisition block (indicated by red dashed lines on Figures 3–4). All other peaks are “off‐diagonal” peaks.

### 2.3 Coherence Selective ULF COSY

The COSY experiment with phase cycling solves the problem of overlapping aliased signals. For the selective observation of multiple quantum coherences the experiment was repeated four times with the following phases of two excitation pulses (Scheme 1): $\varphi_{1}$ =*x*, *y*, −*x*, −*y*, $\varphi_{2}$ =4(*x*). Then, four different $\varphi_{rec}$ cycles were used to select different orders, *p*_1_, of quantum coherences during the *t*_1_ interval: (A) $\varphi_{rec}$ =*x*, −*y*, −*x*, *y* selects *p*_1_=1+4*n*; (B) $\varphi_{rec}$ =*x*, *y*, −*x*, −*y* selects *p*_1_=−1+4*n*; (C) $\varphi_{rec}$ =*x*, −*x*, *x*, −*x* selects *p*_1_=2+4*n*; (D) $\varphi_{rec}$ =4(*x*) selects *p*_1_=4*n* with n being an integer number (Figure 3 and Table S4). There are 11 multiple quantum coherences for a 5 spin‐1/2 system: 0, ±1 , ±2 , ±3 , ±4 and ±5 . And all of them can be excited with the first $90_{\;}^{0}$ pulse [(SI, Eq (S3)] if appropriate multiple spin orders are initially populated (SI, Section 5).

### 2.4 Thermally Polarized ULF COSY Spectrum

To demonstrate that higher‐order quantum coherences in ULF COSY spectra are the result of the population of higher‐order spin states and not a coherent evolution of magnetization during the COSY sequence we performed additional simulations. A COSY spectrum of a system with only longitudinal initial polarization, i. e. thermal Zeeman polarization of order *p*=0 and rank *l*=1, showed quantum coherences up to the first order only (Figure 4); SABRE polarized systems exhibit higher QCs (Figures 2 and 3).

## 3 Conclusions

To summarize, we demonstrated the hyperpolarization of homo‐ and heteronuclear, multiple spin states in a SABRE experiment using SQUID‐based ULF NMR by observation of multiple QCs. COSY at ULF enables the simultaneous measurement at different resonance frequencies (i), selection of different QCs by phase cycling (ii) and does not require a very precise flip angle calibration or experimental performance to extract small polarization of high spin‐order from the one big signal (like in 1D experiments) that comprises all spin orders (iii).

Although QCs from zero up to the fifth‐order could theoretically be measured for 3FPy, experimentally we obtained and assigned only QCs in the range from −3 to +3 within a single experiment or selectively using a phase cycling scheme. As the simulations showed, the signal intensities of higher‐order QCs are dropping rapidly and are below the noise level.

We believe that this contribution is an important experimental confirmation of a known and underestimated evidence that at the low magnetic field the polarization distributes among all strongly‐coupled spins even if the direct source‐target nuclear spin coupling is small. Moreover, the demonstration that multiple quantum coherences are hyperpolarized with SABRE is important information for continuous improvement of the SABRE performance, but it may be even more important for some exotic methods such as ultra‐low field magnetometers^63^ and RASER.^13^ MR at ULF is a quickly developing MR topic^64^ and this work puts a bridge to 2D ULF spectroscopy, whose role at high‐resolution NMR cannot be overestimated.

## Experimental Section

### Hardware

**Scheme 2:**
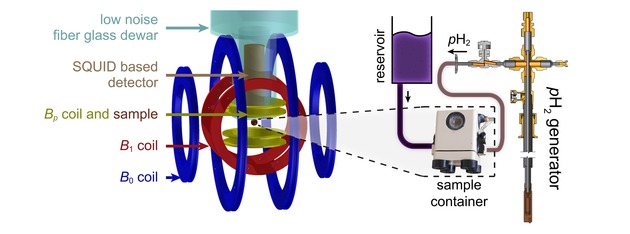
Scheme of the ULF SQUID based MR setup for the hyperpolarization of multiple quantum coherences by SABRE. Freshly produced *p*H_2_ was supplied to the sample container inside a magnetic shield, where the field was varied between the polarizing field *B_p_* and the measurement field *B*_0_. For details, see Ref. [65].

In essence the SQUID‐NMR spectrometer consists of a coil generating the static magnetic field, *B*_0_, a Helmholtz coil to excite the spins by pulses, *B*_1_, a polarizing coil to generate the (elevated) field for SABRE, *B_p_*, and a SQUID‐based detector, which is positioned inside a low noise fiberglass Dewar (Scheme 2). The polarizing coil enables fast switching (≈10 ms) between *B_p_* and *B*_0_ and is placed inside a three‐layered μ‐metal shield. For the experiments, *B_p_* is set to the optimal strength for the SABRE hyperpolarization of ^19^F of the ligands, 5.2 mT,^35^ and reduced to *B*_0_=91 μT for observation of the free induction decay (FID) (see Scheme 1a and Figure S1). 1D and 2D NMR spectra are obtained by 1D and 2D Fourier transformation of the corresponding FIDs. The setup is described in detail in Ref. [65].

### Sample Preparation

Experiments were carried out using two different samples. **Sample 1** contained 29.6 μl 3‐fluoropyridine (3FPy) and 10.5 mg of the [IrIMesCODCl]^56^ self‐synthesized according to Ref. [66] (IMes=1,3‐bis (2,4,6‐trimethylphenyl) imidazole‐2‐ylidene, COD=cyclooctadiene). Both substances were dissolved in 15 ml methanol. **Sample 2** was prepared using 48.7 μl ethyl 5‐fluoronicotinate (EFNA) and the same amount of the [IrIMesCODCl] (10.5 mg) and methanol (15 ml). Results obtained with **Sample 1** are reported in the main text, while those of **Sample 2** are in SI. All samples were filled into a 2 ml sample container that was held at room temperature at the isocenter of the SQUID‐based MR system under atmospheric pressure. This container was connected to a reservoir filled with the rest of the sample (Scheme 1). *p*H_2_ was bubbled continuously through the sample container at a rate of ≈42 scm^3^/min. The experiment was carried out for about 8 hours until ≈13 ml of the solution was evaporated as monitored by MR (Figure S9). The initial concentrations of the Ir‐catalyst and of the fluorinated ligand were 1 mM and 23 mM, respectively.

### Computational Methods

To analyze the experimental results, density matrix simulations were performed using the code of the Magnetic resonance Open source INitiative (MOIN)^[67][27]^ in the following steps:

1. Setting up a spin system of the non‐polarized substrate: four ^1^H, one ^19^F for 3FPy or three ^1^H, one ^19^F for EFNA;
2. Additions of two singlet‐state hydride protons (*p*H_2_) to the system forming the polarized Ir‐complex: IrHH‐3FPy or IrHH‐EFNA, where IrHH represents the hydride protons of transient Ir‐complex;
3. Removal of coherences of the density matrix written in the Eigen basis of the systems Hamiltonian at $B_{p}$ (polarization transfer in Ir‐complex);
4. Removal of the two hydride protons from the system (dissociation of the substrate);
5. Application of the pulse sequence to the free substrate (3FPy or EFNA), dissolved H_2_ and IrHH‐3FPy or IrHH‐EFNA complex. The Liouville von Neuman equation was used to evolve the spin system.

J‐coupling constants for 3FPy and EFNA are listed in Tables S1 and S2.

To investigate the effect of pulse sequences on the Ir‐complex, step 4 (dissociation) was omitted. J‐coupling constants were taken from literature or were estimated,^35^ excitation pulses were treated as ideal rotations with zero duration (hard pulses), and spin relaxation was neglected. More details and an analytical description of the sequence performance is given in the supplementary materials (SI, Sections 2–4**)**. The code for obtaining the simulated COSY spectra is provided via Ref. [66].

## Supporting Information

NMR parameters of 3FPy and EFNA, additional materials to ULF SABRE experiments, measurement of signal stability during the long lasting experiments, evaluation of QCs frequencies and used phase cycling schemes (.PDF)

## Conflict of interest

The authors declare no conflict of interest.

## Supporting information

As a service to our authors and readers, this journal provides supporting information supplied by the authors. Such materials are peer reviewed and may be re‐organized for online delivery, but are not copy‐edited or typeset. Technical support issues arising from supporting information (other than missing files) should be addressed to the authors.

Supplementary
