# Supplementary material for: Multiple Quantum Coherences Hyperpolarized at Ultra‐Low Fields
Source: Chemphyschem. 2019 Oct 17;20(21):2823–9. doi: 10.1002/cphc.201900757 (PMC6900040; doi:10.1002/cphc.201900757)
Supplement: Supplementary file 1 — Supplementary [file CPHC-20-2823-s001.pdf]

CHEMPHYSCHEM

# Supporting Information

© Copyright Wiley-VCH Verlag GmbH & Co. KGaA, 69451 Weinheim, 2019

## Multiple Quantum Coherences Hyperpolarized at Ultra-Low Fields

Kai Buckenmaier,* Klaus Scheffler, Markus Plaumann, Paul Fehling, Johannes Bernarding, Matthias Rudolph, Christoph Back, Dieter Koelle, Reinhold Kleiner, Jan-Bernd Hövener, and Andrey N. Pravdivtsev*© 2019 The Authors. Published by Wiley-VCH Verlag GmbH & Co. KGaA.
This is an open access article under the terms of the Creative Commons Attribution License, which permits use, distribution and reproduction in any medium, provided the original work is properly cited.

# Contents

1. NMR parameters of 3FPy and EFNA .......... S-2
2. Pulse sequence parameters .......... S-3
3. $^{1}H$-$^{19}F$-double frequency excitation pulse .......... S-5
4. FAFOS: additional results .......... S-6
5. COSY: additional results .......... S-9
6. Stability of the SABRE reaction .......... S-13
7. COSY resonance frequencies .......... S-14
8. References .......... S-15

# 1. NMR parameters of 3FPy and EFNA

**Table S1**. J-coupling constants in Hz of 3FPy and estimated coupling of 3FPy with IrHH protons ($IrH^a$ and $IrH^b$). $T_1$-relaxation times are estimated or measured at the high magnetic field and used for simulations. For simplicity, we assume that the chemical shift of 3FPy in the Ir-complex and in the solution does not change. Gyromagnetic ratios are taken from Bruker Almanac 2011 [1].

| 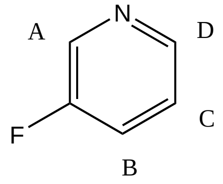 | $IrH^a$ | $IrH^b$ | A | B | C | D | F |
|---|---|---|---|---|---|---|---|
| $IrH^b$ | -7 | | | | | | |
| A | 1.2 | 0 | | | | | |
| B | 0 | 0 | 2.8 | | | | |
| C | 0 | 0 | 0.7 | 8.6 | | | |
| D | 0 | 0.5 | 0 | 1.2 | 4.6 | | |
| F | 1 | 0 | 1 | 8.75 | 4.9 | 1.7 | |
| δ, ppm | -22 | -22 | 8.47 | 7.65 | 7.49 | 8.40 | -127.72 |

**Table S2**. J-coupling constants in Hz of EFNA and estimated coupling of EFNA with IrHH protons ($IrH^a$ and $IrH^b$). $T_1$-relaxation times are estimated or measured at the high magnetic field and used for simulations. For simplicity, we assume that the chemical shift of EFNA in complex and in bulk does not change. Gyromagnetic ratios are taken from Bruker Almanac 2011 [1].

| 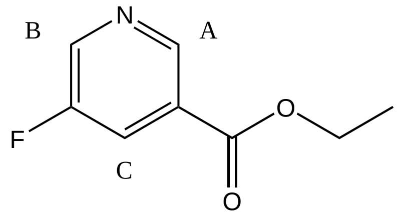 | $IrH^a$ | $IrH^b$ | A | B | C | F |
|---|---|---|---|---|---|---|
| $IrH^b$ | -7 | | | | | |
| A | 1.2 | 0 | | | | |
| B | 0 | 0.5 | - | | | |
| C | 0 | 0 | 1.62 | 2.93 | | |
| F | 1 | 0 | 1.60 | 0.75 | 8.76 | |
| δ, ppm | -22 | -22 | 8.99 | 8.70 | 8.14 | -127.72 |

## 2. Pulse sequence parameters

The sequence parameters are listed in **tab. S3**. The meaning of the parameters is illustrated in **fig. S1**. During the whole sequence, $p\mathrm{H}_2$ was bubbled through the sample with a rate of ≈ 42 $\mathrm{scm}^3$/min and $B_0$ was kept at 91.18 µT. Bp was switched between 0 and 5.2 mT. In **tab. S3** also the frequencies of the $B_1$ excitation pulses are listed. For determining the $B_0$ magnetic field strength and corresponding $^1$H NMR frequency, the resonance frequency of the central (methanol) peak of the $^1$H signal was used.

**FAFOS.** For the Flip Angle FOurier Series (FAFOS) sequence, the flip angle was varied from $0^o$ to $360^o$ with a step size of approx. $3.6^o$. Altogether $N$ = 101 1D spectra with variable $\varphi$ were acquired. After that, a Fourier series along the $\varphi$-dimension was calculated (**eq. S2**). It has to be mentioned that the flip angle was determined from a calibration measurement for a $B_1$ pulse exciting only $^1$H or $^{19}$F. The excitation pulse for both nuclei was difficult to calibrate for two reasons. The first is the influence of the multiple quantum coherences. And the second is crosstalk due to a not small enough excitation bandwidth of the two frequency components of the pulse. The total time for the experiment was ~ 1.5 h.

**COSY.** For the COSY sequences, the hyperpolarization time was kept as short as possible in order to reduce TR, by the cost of lower signal intensity. A short TR enables more measurement steps, as a result, higher spectral resolution and broader spectral width in the indirect $t_1$-encoding dimension is achieved.

**COSY 3FPy**. The measurement of the COSY spectrum for 3FPy without phase cycling (PC) was separated into 18 blocks. Each block contains 400 of constantly incremented $t_1$ steps with $\Delta t_1$ = 0.25 ms, which corresponds to a spectral width (SW) of 4 kHz in the indirect $t_1$-dimension of the COSY spectrum. An overall record of 18 blocks allowed us to vary $t_1$ from 20 ms up to 1820 ms, which corresponds to a spectral resolution of 0.6 Hz. After each block, a control spectrum was acquired.

**COSY EFNA**. The measurement of the COSY spectrum for EFNA without PC was separated into 20 blocks. Each block contains 200 of constantly incremented $t_1$ steps with $\Delta t_1$ = 0.5 ms, which corresponds to SW = 2 kHz in the indirect $t_1$-dimension of the COSY spectrum. An overall record of 20 blocks allowed us to vary $t_1$ from 20 ms up to 2020 ms, which corresponds to a spectral resolution of 0.5 Hz. After each block, a control spectrum was acquired.

**COSY PC 3FPy**. The measurement of the COSY spectrum with PC for 3FPy was separated into 19 blocks. Each block contains 80 of constantly incremented $t_1$ time steps. As for the previous measurement, after each block, a control measurement was performed. Since each $t_1$ time step needs 4 phase cycling steps the SW and resolution were lower as for the measurement without phase cycling. $\Delta t_1$ was set to 0.5 ms resulting in SW = 2 kHz and a spectral resolution of 1.3 Hz.

**COSY PC EFNA**. The measurement of the COSY spectrum with PC for EFNA was separated into 14 blocks. Each block contains 80 of constantly incremented $t_1$ time steps. After each block, a control measurement was performed. $\Delta t_1$ was set to 0.5 ms resulting in SW = 2 kHz and a spectral resolution of 1.8 Hz.

**Table S3.** Sequence parameters for the FAFOS and the COSY sequences for the measurements shown. The parameters are illustrated in fig. SISEQ

| | 3FPy | | | EFNA | | |
|---|---|---|---|---|---|---|
| | **FAFOS** | **COSY** | **COSY PC** | **FAFOS** | **COSY** | **COSY PC** |
| $t_{ramp}$ **[ms]** | 30 | 30 | 30 | 30 | 30 | 30 |
| $t_{Bp}$ **[s]** | 4 | 2 | 2 | 4 | 2.5 | 2.5 |
| $t_w$ **[ms]** | 11 | 11 | 11 | 11 | 11 | 11 |
| $t_{B1}$ **[ms]** | 15 | 15 | 15 | 20 | 15 | 15 |
| $t_{B1,w}$ **[ms]** | 7 | 7 | 4 | 7 | 7 | 7 |
| $t_{acq}$ **[s]** | 8 | 2 | 2 | 8 | 2 | 2 |
| **TR [ms]** | 12150 | 4300 – 5900 | 4169 – 4829 | 12160 | 4572 – 6572 | 4572 – 5132 |
| $t_1$ **[ms]** | — | 20 – 1720 | 20 – 780 | — | 20 – 2020 | 20 – 580 |
| **steps** | 141 | 6801 | 1521 | 241 | 4001 | 1121 |
| **avg.** | 2 | 1 | 4 | 2 | 1 | 4 |
| $B_1$ freq $^1$H [Hz] | 3880 | 3875 | 3875 | 3880 | 3880 | 3880 |
| $B_1$ freq $^{19}$F [Hz] | 3650 | 3650 | 3650 | 3650 | 3650 | 3650 |
| $^1$H NMR freq [Hz] | 3882.5 | 3877.9 | 3881.1 | 3882.0 | 3881.9 | 3882.0 |

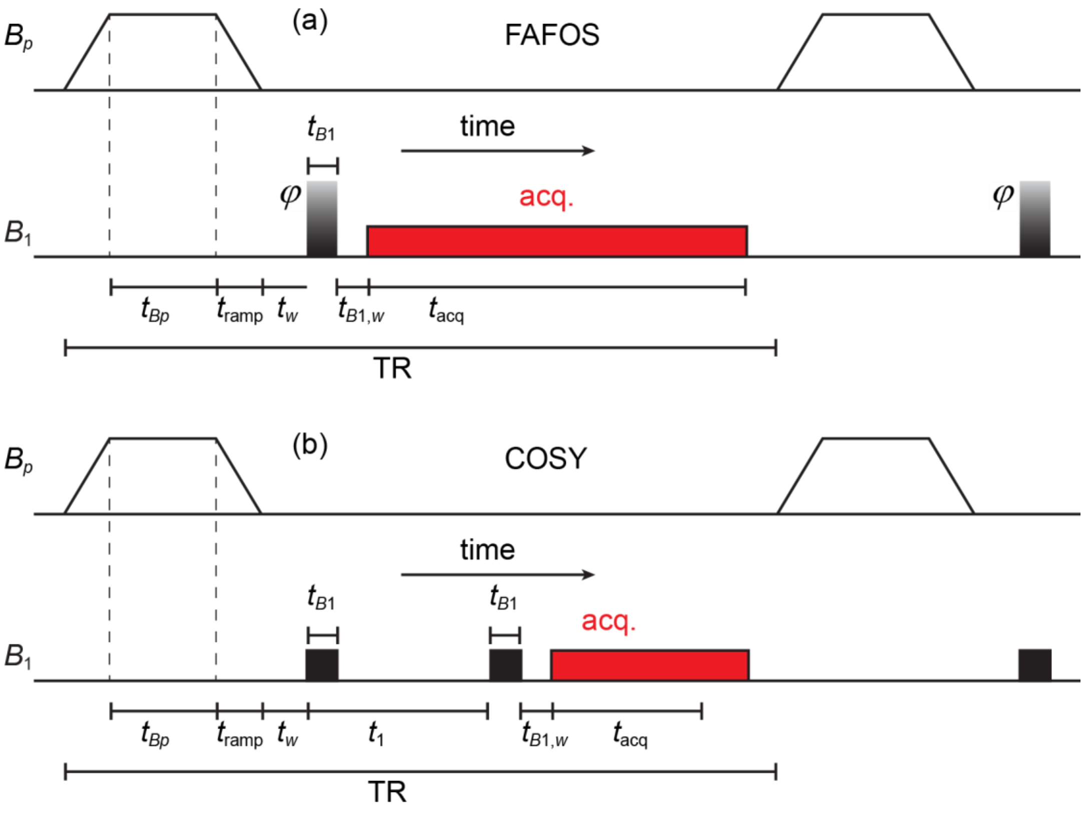


**Figure S1.** Illustration of sequence parameters for (a) the FAFOS and (b) the COSY sequences.

## 3. $^1$H-$^{19}$F-double frequency excitation pulse

The $B_1$ excitation pulse can be described by the expression:

$$f(t) = (\sin(\omega_{1H}t) + \sin(\omega_{19F}t)) \cdot \mathrm{sinc}(4 \cdot (t - t_{\mathrm{end}}/2)/t_{\mathrm{end}}),$$

Here $t_{\mathrm{end}}$ is the length of the total pulse and $\omega_{1H}$ and $\omega_{19F}$ are the excitation frequencies. An excitation pulse used for the experiments is shown in **fig. S2 (a)**. **Figure S2 (b)** shows the single-sided Fourier spectrum $F(\omega)$ of the pulse.

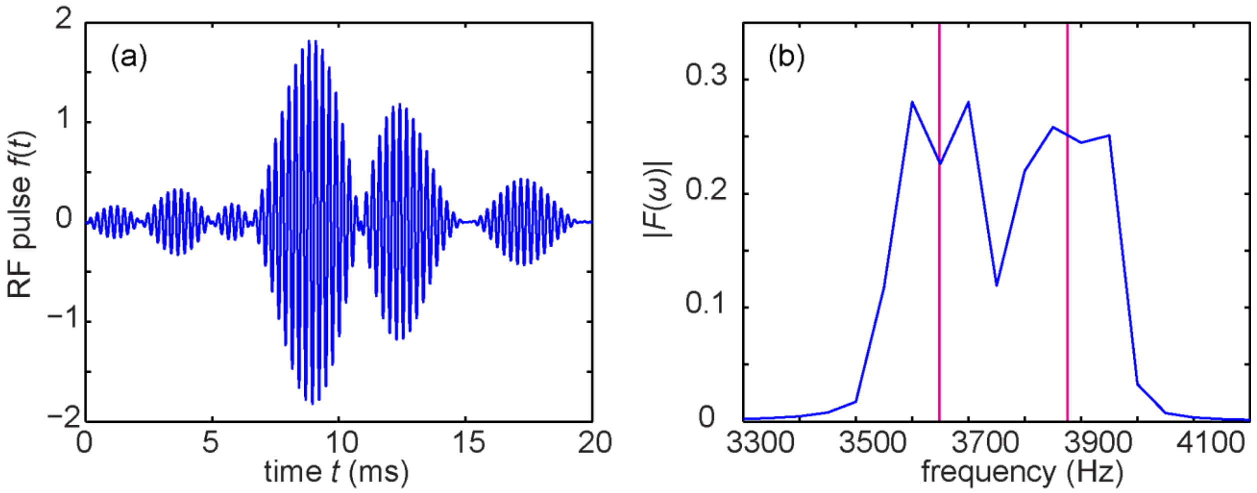


**Figure S2**. Sinc shaped modulated excitation pulse (a) and the corresponding amplitude spectrum (b). The excitation profile allows simultaneous excitation at two frequencies. At $B_0$ = 91.18 µT resonance frequencies of $^1$H and $^{19}$F are ~ 3880 Hz and ~ 3650 Hz respectively (marked on (b)). Similar excitation profiles were used in the experiments shown in the manuscript. Note that because the two excitation regions are overlapping and have different intensities the experimental excitation flip angle could deviate from the nominal value by 5 – 10%.

## 4. FAFOS: additional results

FAFOS (**fig. S1 (a)**) [55–57] (Flip Angle FOurier Series) was used to reveal hyperpolarized high-order spin states. FAFOS is a simple 1D sequence where the flip angle $\varphi$ is varied.

If the full density matrix approach is used to calculate the spin state of a SABRE system at ULF [2,3], it is easy to show that not only longitudinal magnetization but also collective spin orders with total zero-quantum coherence are populated.

For an *N* spin-½ system, the longitudinal magnetization is represented by *N* spin operators. Since we are interested e.g., in 3FPy, hereafter a five spin system with four longitudinal [1]H operators $\hat{T}_Z = \hat{I}_Z$, $\hat{S}_Z$, $\hat{R}_Z$, $\hat{K}_Z$ and one [19]F operator $\hat{T}_Z$ = $\hat{F}_Z$ is discussed. These operators represent the spins of the nuclei A, B, C, D and F of 3FPy (**tab. S1**, **SI**).

The collective spin orders of a total zero-quantum coherence are represented by ten $\hat{T}_{ZZ}$ terms (e.g., $\hat{I}_Z\hat{R}_Z$), ten $\hat{T}_{3Z}$, terms (e.g., $\hat{I}_Z\hat{S}_Z\hat{R}_Z$), five $\hat{T}_{4Z}$ terms (e.g., $\hat{I}_Z\hat{S}_Z\hat{R}_Z\hat{T}_Z$), one $\hat{T}_{5Z}$ = $\hat{I}_Z\hat{S}_Z\hat{R}_Z\hat{T}_Z\hat{F}_Z$ term and more other spin orders that are combinations of zero-quantum coherences with various $\hat{T}_Z$ terms (e.g., $\hat{I}_X\hat{S}_X\hat{F}_Z + \hat{I}_Y\hat{S}_Y\hat{F}_Z$). It is very difficult to distinguish between different spin orders by 1D high-resolution NMR spectroscopy. At ULF the conditions are even more complicated, because of a negligible chemical shift all nuclei of one type have the same resonance frequency.

The first approach we discussed in the main text is FAFOS. When a $B_1$ pulse is applied to a system that is described by one of the $\hat{T}_Z$ terms the observed signal is proportional to $\sin(\varphi)$ and the "$-1$" quantum coherence ($\hat{T}_{-1}$) is observable. When a system is described by other spin orders, the observed signal amplitudes are proportional to the functions:

$$
\begin{gathered}
\hat{T}_Z \xrightarrow{\varphi} \sin(\varphi)\,\hat{T}_{-1} \\
\hat{T}_{ZZ} \xrightarrow{\varphi} \sin(\varphi)\cos(\varphi)\,\hat{T}_{-1} = \frac{1}{2}\sin(2\varphi)\,\hat{T}_{-1} \\
\hat{T}_{3Z} \xrightarrow{\varphi} \sin(\varphi)\cos^2(\varphi)\,\hat{T}_{-1} = \\
= \frac{1}{4}(\sin(\varphi) + \sin(3\varphi))\hat{T}_{-1} \\
\hat{T}_{4Z} \xrightarrow{\varphi} \sin(\varphi)\cos^3(\varphi)\,\hat{T}_{-1} = \\
= \frac{1}{8}(2\sin(2\varphi) + \sin(4\varphi))\hat{T}_{-1} \\
\hat{T}_{5Z} \xrightarrow{\varphi} \sin(\varphi)\cos^4(\varphi)\,\hat{T}_{-1} = \\
= \frac{1}{16}(2\sin(\varphi) + 3\sin(3\varphi) + \sin(5\varphi))\hat{T}_{-1}
\end{gathered}
\qquad \textbf{(eq S1)}
$$

The amplitude of the different $\sin(n\varphi)$ terms can be obtained by evaluating the Fourier coefficient series (FCs), $c_k(\omega)$, of the spectra $S(\omega,\varphi)$ in the direction of the flip angle sweep with

$$c_k(\omega) = \sum_{j=1}^{L} S(\omega,\varphi_j)\sin(k\varphi_j) \qquad \textbf{(eq S2)}$$

where $k$ is a positive integer number, $\varphi_j = \frac{2\pi}{L}j$ is the flip angle and *L* is given by $\varphi_L = 2\pi$.

Hence, this method perfectly separates $\hat{T}_Z$ from $\hat{T}_{ZZ}$, if no other terms are present. However, all odd and even $\hat{T}_{nZ}$ terms have some common $\sin(n\varphi)$ functions, e.g., for $\hat{T}_Z$, $\hat{T}_{3Z}$ and $\hat{T}_{5Z}$ it is $\sin(\varphi)$. Therefore, a simple Fourier series along the flip angle sweep used before will not separate different spin orders and additional post-processing is necessary. Although this method can be used for a

demonstration of the presence of higher spin orders, a quantitative analysis is difficult. The presence of zero-quantum coherences complicates the situation and is not described by **eq. S1**.

Calculations predict that at least the first three FCs should be observable. However, it is challenging experimentally to obtain higher harmonics, because a higher-order series has a lower intensity, all FCs contribute to the one signal at a given frequency and polarization level deviate a little with time. Especially the contribution of non-perfect excitation pulses makes a quantitative analysis nearly impossible (**fig. S2**).

The comparison of experimental and simulated FCs for $^{1}H$ and $^{19}F$ is shown in **fig. S3** and **S4**. The presence of components of a Fourier series along flip angle sweep showed the presence of high-order spin states. However, quantitative analysis with this method proved difficult; the overall amplitudes of the first three Fourier components had similar relative intensities compared to simulations, in which higher-order spin states were populated. The phase and amplitude of each peak could not be correlated and as a result, the influence of different hyperpolarized spin orders was not identified. The simulations revealed that almost perfect hard excitation pulses are necessary for such a quantitative analysis – experimentally, this was difficult to realize. Because of the limited excitation bandwidth, the excitation of the nuclei varied by 5 – 10 % (**SM section 3**). This was the case for both substrates, 3FPy and EFNA (**fig. S3**, **S4**).

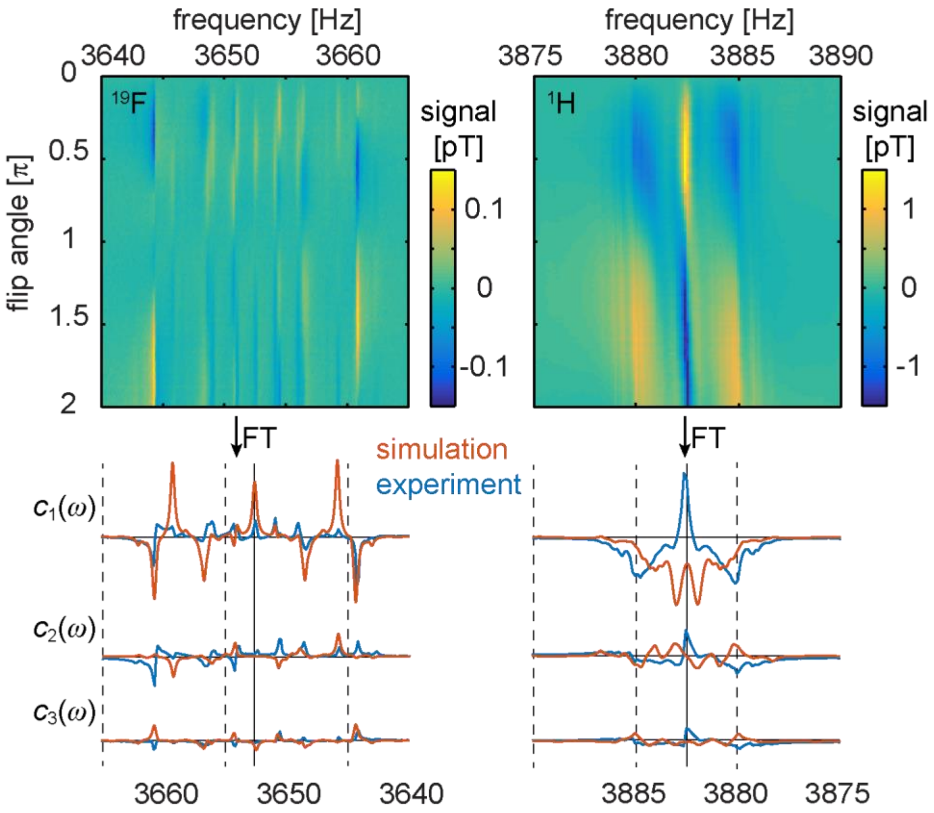


**Figure S3**. ULF SABRE FAFOS spectra $S(\omega, \varphi)$ (upper part) and comparison of experimental (blue lines) and simulated (red lines) FCs, $c_{1,2,3}(\omega)$, (lower part) for $^{19}F$ (left side) and $^{1}H$ of 3FPy. The black symmetry line indicates the centre frequency of the $^{19}F$ and $^{1}H$ signal. J-coupling constants for 3FPy are listed in **tab. S1**.

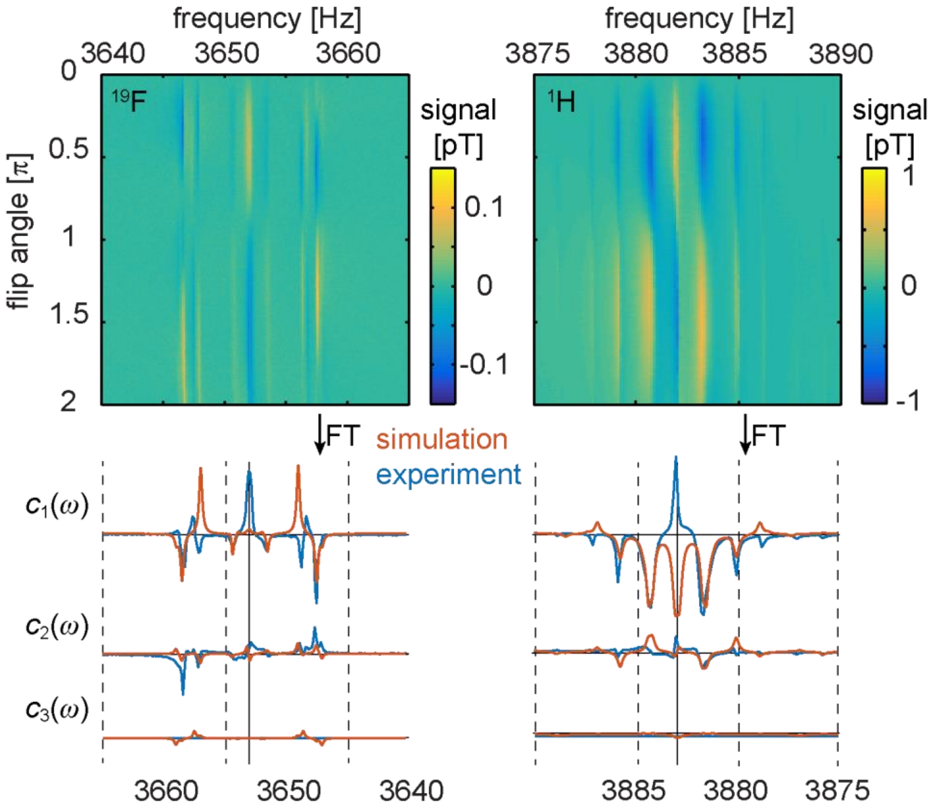


**Figure S4**. ULF SABRE FAFOS spectra (upper part) and comparison of experimental (blue lines) and simulated (red lines) FCs, $c_{1,2,3}(\omega)$, (lower part) for$^{19}$F (left side) and $^{1}$H of EFNA. The black symmetry line indicates the centre frequency of the $^{19}$F and $^{1}$H signal. The simulation parameters are $B_0$ = 91.18 µT, $B_p$ = 5.2 mT, J-coupling constants for 3FPy are listed in **tab. S2**.

## 5. COSY: additional results

An alternative method to FAFOS discussed in the main text is based on a conventional COSY experiment. After the first $90^0_{\phi 1}$ pulse the $\hat{T}_Z$ terms transfer to the combination of single quantum coherences $\hat{T}_{\pm 1}$, i.e., $\hat{I}_Z \xrightarrow{90^o_Y} \hat{I}_X = (\hat{I}_+ + \hat{I}_-)/2$, or more generally, $\hat{T}_Z \xrightarrow{90^o} \hat{T}_{\pm 1}$. The transformation of other collective spin orders discussed can be shown in the same fashion and results in:

$$\begin{gathered} \hat{T}_Z \xrightarrow{90^o} \hat{T}_{\pm 1} \\ \hat{T}_{ZZ} \xrightarrow{90^o} \hat{T}_{\pm 2}, \hat{T}_0 \\ \hat{T}_{3Z} \xrightarrow{90^o} \hat{T}_{\pm 3}, \hat{T}_{\pm 1} \\ \hat{T}_{4Z} \xrightarrow{90^o} \hat{T}_{\pm 4}, \hat{T}_{\pm 2}, \hat{T}_0 \\ \hat{T}_{5Z} \xrightarrow{90^o} \hat{T}_{\pm 5}, \hat{T}_{\pm 3}, \hat{T}_{\pm 1} \end{gathered} \qquad \text{(eq S3)}$$

As for the FAFOS method, the odd and even terms here have common resulting coherences. Hence, although COSY spectroscopy at ULF cannot directly separate different $\hat{T}_{nZ}$ terms, it allows a selective observation of high order quantum coherences (**fig. 2**, and **fig. S5**).

A possible phase cycle of the COSY experiment (**fig. S2(b)**) is as follows: $\phi_1$ = x, y, -x, -y, $\phi_2$ = 4(x), $\phi_{rec}$ = x, -y, -x, y. The variation of $\phi_{rec}$ – cycle results in 4 different COSY spectra (**tab. S4, A-D**). At the high magnetic field, usually one out of four phase-cycling schemes is used to select the single quantum coherence, $p_1 = -1$, during the interpulse delay, $t_1$. However, other coherences, namely $p_1 = -1 + 4n = -5, -1, 3, \ldots$ with $n$ being an integer number, are also selected but usually are neglected.

All four variants of the phase-cycling filtering together with coherence selection pathways are listed in **tab. S4** and performance are demonstrated on **fig. 3** and **S6**. Altogether there are 11 multiple quantum coherences for a 5 spin-1/2 system: $0$, $\pm 1$, $\pm 2$, $\pm 3$, $\pm 4$ and $\pm 5$. And all of them can be excited with the first $90^0_{\phi 1}$ pulse (**eq. S3**) if appropriate spin orders are populated.

**Table S4**. Four COSY multi-quantum coherence selection phase cycling schemes: (A) $p_1 = -3$, 1, 5, (B) $p_1 = -5$, $-1$, 3, (C) $p_1 = \pm 2$, (D) $p_1 = 0$, $\pm 4$. $\phi_{\mathrm{rec}}$ coincides with the total phase of the pulse sequences and provides a selection of the $p_1$ quantum coherence.

| Case / step | $\phi_1$ | Phase for $p_1 - p_0$ | $\phi_2$ | Phase for $p_2 - p_1$ | Total phase $\phi_{rec}$ |
|---|---|---|---|---|---|
| **A** | $p_1 = -3, 1, 5;\ \ 1 + 4n$ | | | | |
| 1 | 0° | 0° | 0° | 0° | 0° |
| 2 | 90° | 270° | 0° | 0° | 270° |
| 3 | 180° | 180° | 0° | 0° | 180° |
| 4 | 270° | 90° | 0° | 0° | 90° |
| **B** | $p_1 = -5, -1, 3; -1 + 4n$ | | | | |
| 1 | 0° | 0° | 0° | 0° | 0° |
| 2 | 90° | 90° | 0° | 0° | 90° |
| 3 | 180° | 180° | 0° | 0° | 180° |
| 4 | 270° | 270° | 0° | 0° | 270° |
| **C** | $p_1 = \pm 2;\ 2 + 4n$ | | | | |
| 1 | 0° | 0° | 0° | 0° | 0° |
| 2 | 90° | 180° | 0° | 0° | 180° |
| 3 | 180° | 0° | 0° | 0° | 0° |
| 4 | 270° | 180° | 0° | 0° | 180° |
| **D** | $p_1 = 0, \pm 4;\ \ 4n$ | | | | |
| 1 | 0° | 0° | 0° | 0° | 0° |
| 2 | 90° | 0° | 0° | 0° | 0° |
| 3 | 180° | 0° | 0° | 0° | 0° |
| 4 | 270° | 0° | 0° | 0° | 0° |

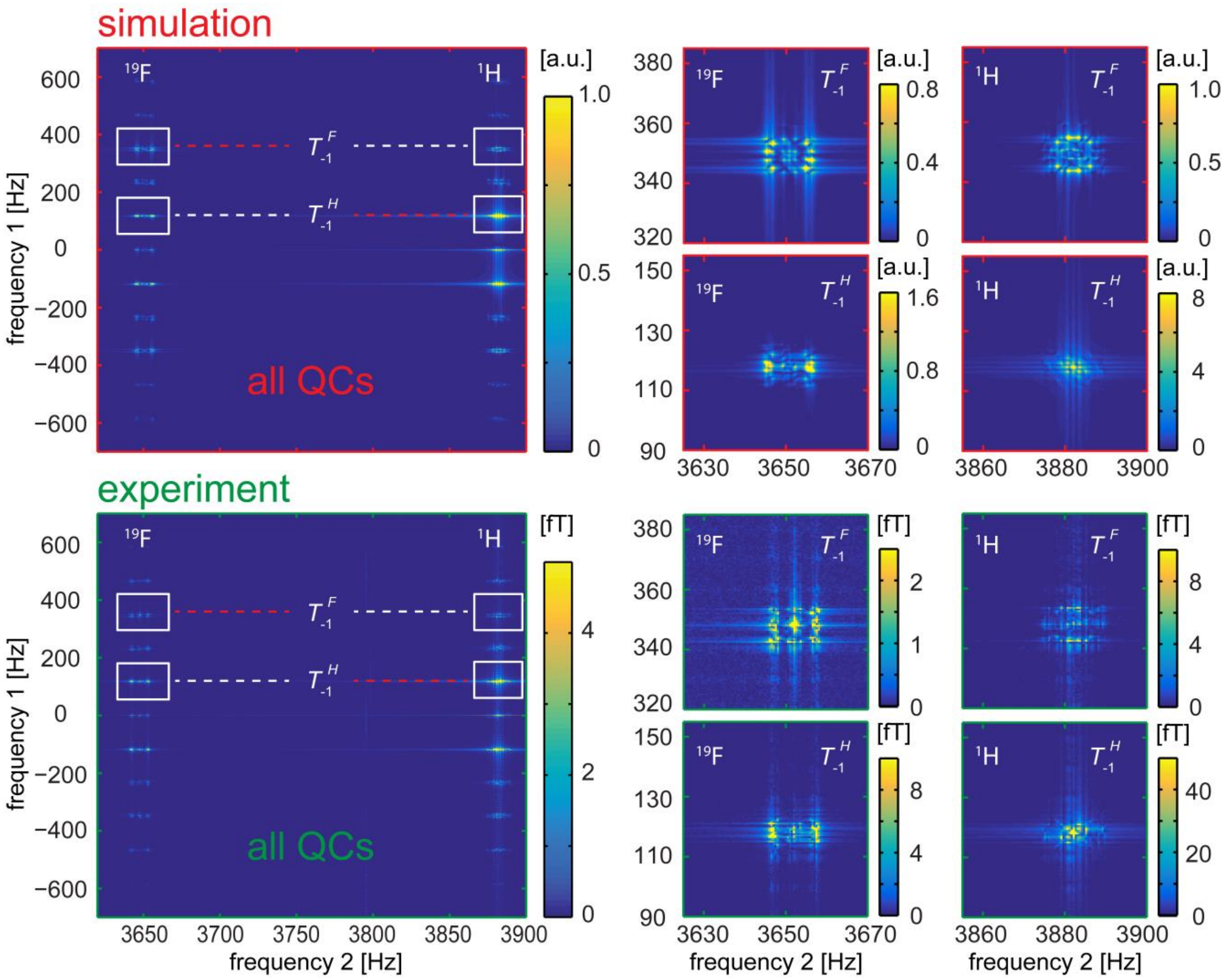


**Figure S5.** Experimental (lower half) and simulated (upper half) ULF SABRE COSY amplitude spectra of EFNA. On the right, the zoomed out $T_{-1}^{H}$ and $T_{-1}^{F}$ QCs measured by $^{1}$H and $^{19}$F are shown (indicated by the white rectangles). The red and white dashed lines mark the diagonal and off-diagonal peaks respectively. The simulation parameters are $B_0$ = 91.18 µT, $B_p$ = 5.2 mT, J-coupling constants for EFNA are listed in **tab. S2**.

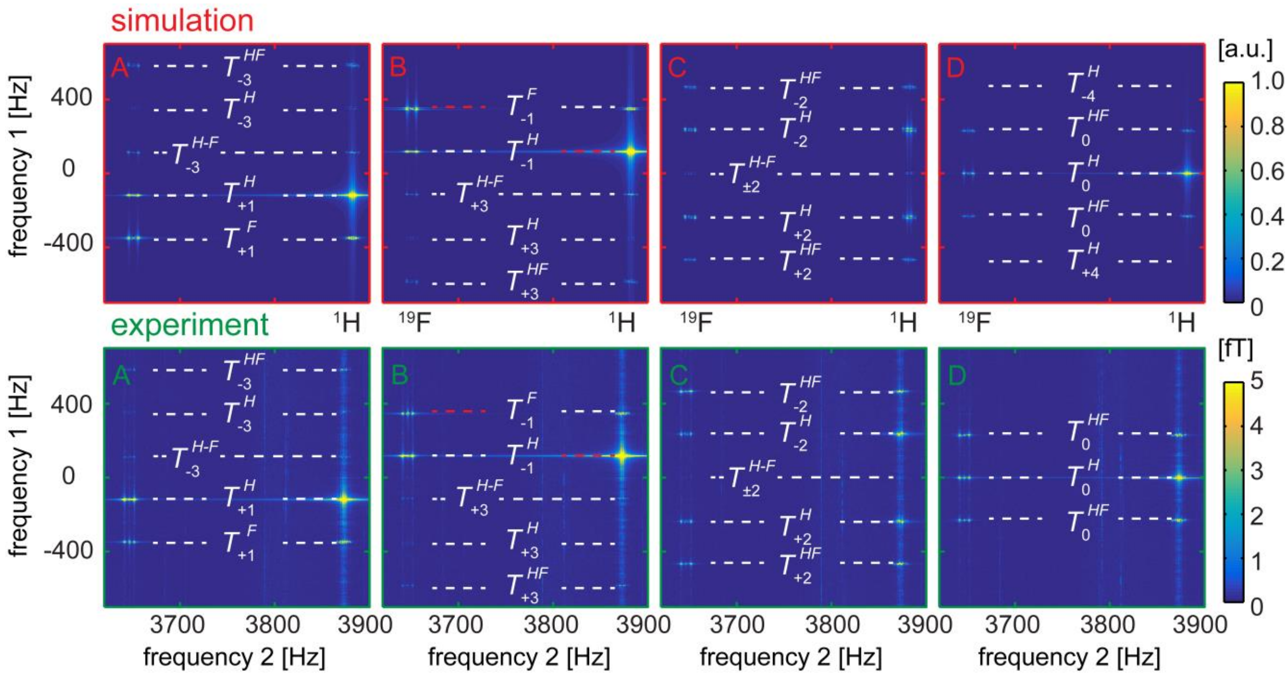


**Figure S6.** Simulated (top) and experimental (bottom) EFNA COSY amplitude spectra obtained with four different phase alternating methods (here A-D corresponds to phase cycling schemes given in **tab. S4**). The QCs, T, are assigned using **tab. S5**. The red and white dashed lines mark the diagonal and off-diagonal peaks, respectively. The simulation parameters are $B_0$ = 91.18 µT, $B_p$ = 5.2 mT, J-coupling constants for EFNA are listed in **tab. S2**.

## 6. Stability of the SABRE reaction

During all measurements, control spectra were acquired. The area under the absolute value of the $^1$H peaks was integrated as a reference value for the SABRE enhancement factor. The time course is shown on **fig. S7**. The drop off at the end of each curve indicates that the level of the sample container was lowered and that the reservoir was empty.

The sequence parameters for all spectra were the same. The hyperpolarization time $t_{Bp}$ was set to 8 s and $t_{acq}$ to 8 s. The other parameters were similar to the parameters listed in **tab. S3**.

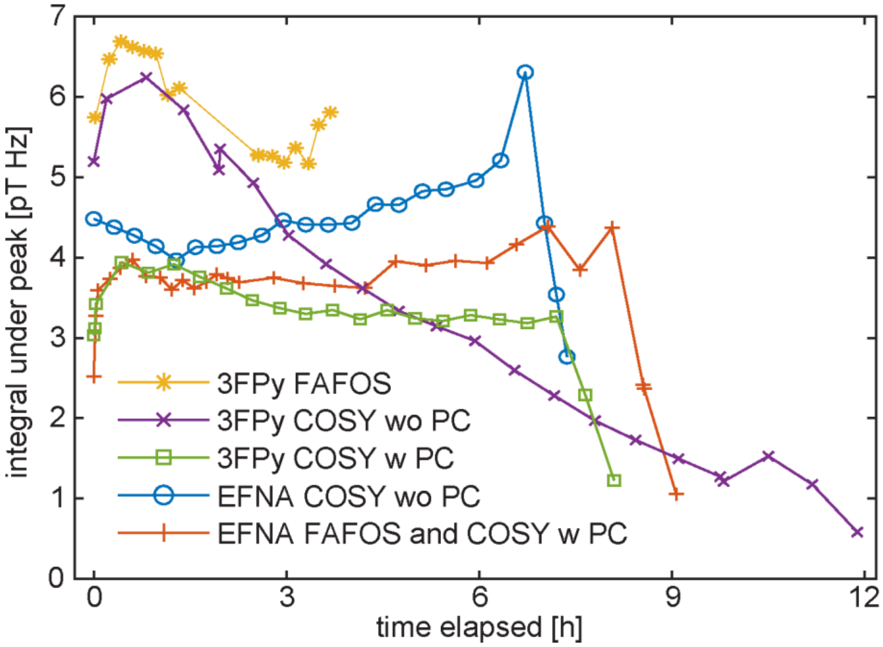


**Figure S7.** Integral under the absolute value of the $^1$H peaks as a function of time during the measurements.

## 7. COSY resonance frequencies

Spin rotation under the action of an external static magnetic field is given by the following two equations [4]:

$$\hat{I}_X \xrightarrow{-\tau\omega\hat{I}_Z} \hat{I}_X \cos(\omega\tau) - \hat{I}_Y \sin(\omega\tau)$$

$$\hat{I}_Y \xrightarrow{-\tau\omega\hat{I}_Z} \hat{I}_Y \cos(\omega\tau) + \hat{I}_X \sin(\omega\tau)$$

Since $\pm 1$ QCs, $\hat{T}_\pm$, equal to $\hat{I}_\pm = \hat{I}_X \pm i\hat{I}_Y$. Then their evolution is described as follows:

$$\hat{I}_\pm \xrightarrow{-\tau\omega\hat{I}_Z} \hat{I}_\pm e^{\pm i\tau\omega} \quad \textbf{(eq S4)}$$

From this, it follows that $T_{\pm 1}^{X}$ QCs evolve with $\pm\omega_X$ frequencies respectively. Using **eq. S4** one can obtain evolution frequencies of all other QCs that are listed in **tab. S5**.

**Example 1**. $T_0^{HF}$ QCs are a product of one $T_{+1}^{H}$ and one $T_{-1}^{F}$ or $T_{-1}^{H}$ and one $T_{+1}^{F}$, therefore according to **eq. S4**, $T_0^{HF}$ evolves with the frequencies $\pm(\omega_{1H} - \omega_{19F})$ accordingly.

**Example 2**. $T_{+5}^{HF}$ QC is a product of four $T_{+1}^{H}$ QCs and one $T_{+1}^{F}$, therefore according to **eq. S4** $T_{+5}^{HF}$ evolves with the frequency $+(4\omega_{1H} + \omega_{19F})$.

**Example 3**. $T_{+3}^{H-F}$ QC is a product of four $T_{+1}^{H}$ QCs and one $T_{-1}^{F}$, therefore according to **eq. S4** $T_{+5}^{HF}$ evolves with the frequency $+(4\omega_{1H} - \omega_{19F})$.

**Table S5**. Frequencies of multiple quantum coherences from -5 to +5 for a five spin ½ system with four protons and a single $^{19}$F nucleus (3FPy). Upper indexes indicate that only protons (H superscript) or also $^{19}$F (HF superscript) comprises the coherence. Aliased resonance frequencies are given here for the SW of 2 kHz or 4 kHz. Note that coherences $T_n^{H-F}$ are not discussed in the main text because they are expected to have much lower signal intensities than corresponding $T_n^{HF}$.

| QC | Frequency ($B_0$=91.18 μT), Hz | Aliased res., Hz (SW = 2 or 4 kHz) |
|---|---|---|
| $T_0^H$ | $v_{1H} - v_{1H}$=0 | 0 |
| $T_0^{HF}$ | $\pm(v_{1H} - v_{19F})$=±231 | ±231 |
| $T_{\pm1}^H$ | $\pm v_{1H}$=±3882 | ∓118 |
| $T_{\pm1}^F$ | $\pm v_{19F}$=±3651.5 | ∓348.5 |
| $T_{\pm1}^{H-F}$ | $\pm(2v_{1H} - v_{19F})$=±4112.9 | ±112.9 |
| $T_{\pm2}^H$ | $\pm 2v_{1H}$=±7764.4 | ∓235.6 |
| $T_{\pm2}^{HF}$ | $\pm(v_{1H} + v_{19F})$=±7533.7 | ∓466.3 |
| $T_{\pm2}^{H-F}$ | $\pm(3v_{1H} - v_{19F})$=±7995.1 | ∓4.9 |
| $T_{\pm3}^H$ | $\pm 3v_{1H}$=±11647 | ∓353 |
| $T_{\pm3}^{HF}$ | $\pm(2v_{1H} + v_{19F})$=±11416 | ∓584 |
| $T_{\pm3}^{H-F}$ | $\pm(4v_{1H} - v_{19F})$=±11877.4 | ∓122.6 |
| $T_{\pm4}^H$ | $\pm 4v_{1H}$=±15529 | ∓471 |
| $T_{\pm4}^{HF}$ | $\pm(3v_{1H} + v_{19F})$=±15298 | ∓702 |
| $T_{\pm5}^{HF}$ | $\pm(4v_{1H} + v_{19F})$=±19180 | ∓820 |

# 8. References


[1] https://lsa.umich.edu/content/dam/chem-assets/chem-docs/BrukerAlmanac2011.pdf

[2] A. N. Pravdivtsev, A. V. Yurkovskaya, H.-M. Vieth, K. L. Ivanov, and R. Kaptein, ChemPhysChem **14**, 3327 (2013).

[3] S. Knecht, A. N. Pravdivtsev, J.-B. Hövener, A. V. Yurkovskaya, and K. L. Ivanov, RSC Adv. **6**, 24470 (2016).

[4] O. W. Sørensen, G. W. Eich, M. H. Levitt, G. Bodenhausen, and R. R. Ernst, Progress in Nuclear Magnetic Resonance Spectroscopy **16**, 163 (1984).
